# Do Not Respond! Doing the Think/No-Think and Go/No-Go Tasks Concurrently Leads to Memory Impairment of Unpleasant Items during Later Recall

**DOI:** 10.3389/fpsyg.2012.00269

**Published:** 2012-08-01

**Authors:** Cornelia Herbert, Stefan Sütterlin

**Affiliations:** ^1^Department of Psychology, University of WürzburgWürzburg, Germany; ^2^Research Unit INSIDE, University of LuxembourgLuxembourg

**Keywords:** memory suppression, emotion, response inhibition, go/no-go task, think/no-think paradigm

## Abstract

Previous research using neuroimaging methods proposed a link between mechanisms controlling motor response inhibition and suppression of unwanted memories. The present study investigated this hypothesis behaviorally by combining the think/no-think paradigm (TNT) with a go/no-go motor inhibition task. Participants first learned unpleasant cue-target pairs. Cue words were then presented as go or no-go items in the TNT. Participants’ task was to respond to the cues and think of the target word aloud or to inhibit their response to the cue and the target word from coming to mind. Cued recall assessed immediately after the TNT revealed reduced recall performance for no-go targets compared to go targets or baseline cues not presented in the TNT. The results demonstrate that doing the no-think and no-go task concurrently leads to memory suppression of unpleasant items during later recall. Results are discussed in line with recent empirical research and theoretical positions.

## Introduction

Research into motivated forgetting has received increased attention in recent years. One finding from this field of research is that suppression of an unwanted item increases its availability in memory and enhances recall performance of the item during later recall tests (Wegner et al., 1987, 1990; Wegner, 1994; Soetens et al., 2006; Dunn et al., 2009). This ironic memory enhancement effect has recently been challenged by studies examining memory suppression in the think-no-think (TNT) paradigm (for an overview, see Anderson and Levy, 2009).

In the classical TNT paradigm participants are instructed to think or not to think of a target item that has previously been associated with a cue. For instance, individuals learn that the words ordeal and roach are associated and are then instructed to recall the target (roach) when exposed to the cue (ordeal). At the same time, for certain cues they are instructed to inhibit memory retrieval of the associated target word by preventing its content from entering consciousness (Anderson and Levy, 2009). Additional targets not presented during the TNT serve as baseline. Results revealed reduced recall for “no-think” targets compared to “think” or “baseline” targets.

Memory suppression in the TNT might be explained by inhibitory processes (Anderson et al., 2004). Specifically, it has been suggested that inhibitory processes involved in thought suppression during the TNT might be analogues to inhibitory processes involved in motor response inhibition (Menon et al., 2001; Garavan et al., 2002). This suggestion has been supported by a number of neuroimaging studies. These studies found increased activation in fronto-parietal networks involved in executive control and motor response inhibition during no-think trials in the TNT (Anderson et al., 2004; Depue et al., 2007; Levy and Anderson, 2008; Butler and James, 2010). Specially, activation of the right dorsolateral prefrontal cortex (DLPFC) involved in inhibiting prepotent motor responses (Simmonds et al., 2008) has been associated with decreased memory-related neural activity in the hippocampus during no-think trials (Anderson et al., 2004; Depue et al., 2007; Hanslmayr et al., 2009). Studies investigating clinical samples such as individuals with attention deficit/hyperactivity disorder provided further evidence for a relationship between prefrontally mediated motor inhibition and inhibition of memory retrieval in the TNT (Depue et al., 2010; but see Salamé and Danion, 2007). In addition, a number of studies using electroencephalographic (EEG) recordings in combination with the TNT or a motor response inhibition task reported a positive correlation between the N2 event-related potential component elicited during the stop-signal task (SST; Logan and Cowan, 1984) and the N2 event-related potential component elicited during no-think trials in the TNT (Mecklinger et al., 2009). Depue et al. (2010) reported similar correlational results between the TNT and the SST in conjunction with functional imaging methods.

Although these findings suggest a common neural system involved in both motor response inhibition and memory suppression, few studies provide behavioral evidence in favor of a relationship between motor response inhibition and suppression of unwanted memories. Evidence that processes involved in motor control can lead to memory suppression comes from two recent behavioral studies that examined memory retrieval of emotionally valenced or neutral words after motor response inhibition in the SST (Herbert and Sütterlin, 2011) or in combination with the TNT (Tomlinson et al., 2009). Results revealed significantly reduced memory performance for unpleasant words that were presented in stop trials in the SST (Herbert and Sütterlin, 2011). Tomlinson et al. (2009) found that instructing individuals to quickly press a button instead of not thinking leads to similar memory suppression effects as suppressing thought alone.

The results by Herbert and Sütterlin (2011) support an interference effect of motor response inhibition for emotionally valenced items (particularly unpleasant ones) during the stage of memory acquisition and encoding. The findings by Tomlinson et al. (2009) suggest that memory suppression during no-think trials in the TNT could be accounted for by mechanisms other than active inhibition. In this view, memory impairment in the TNT would result from interference during the memory retrieval stage and not from active inhibition during the suppression phase.

There is another line of research that casts doubt on the hypothesis that motor inhibition leads to memory suppression in the TNT. From the perspective of dual competition models of information processing (e.g., Desimone and Duncan, 1995; Pessoa, 2009) instructing individuals not to respond during no-think trials could act as a distractor task that decreases the efficacy of inhibition during no-think trials by directing processing resources away from memory suppression. Using a retrieval induced forgetting (RIF) paradigm, Román et al. (2009) provided supporting evidence for this speculation: in this study, memory suppression during later recall disappeared when participants had to perform an attention demanding concurrent task during instructed forgetting.

The present study uses a modified version of the TNT, which combines the TNT with the go/no-go response inhibition paradigm. Building upon previous research this dual-task version of the TNT should allow us to determine if processes required for response inhibition and thought suppression interfere with each other and if so, how this interference affects active retrieval of unpleasant targets during later recall.

In particular, if response inhibition and thought suppression competed for the same class of cognitive processing resources, as suggested by dual competition models, performance in at least one of the two tasks should be significantly impaired. In this case, doing the no-think and no-go task concurrently could even be expected to result in ironic rebound effects enhancing later recall of the inhibited items. On the other hand, if motor response inhibition and thought suppression do not compete for cognitive processing resources because both tasks are inhibitory in nature, possibly activating a common inhibitory prefrontal control system (e.g., Berkman et al., 2009), target words associated with no-go/no-think trials should be significantly less well remembered during later recall compared to targets associated with go/think trials or target words that were not part of any intervention. Additionally, participants should be able to perform both tasks equally well.

## Materials and Methods

### Participants

Participants were 38 healthy, young adults (15 male; *M* = 23.1 years; SD = 4.2 years). Participants were recruited via advertisement in the local newspaper and the posting board at the University of Würzburg. Exclusion criteria for participation were current and previous psychiatric, neurological, or somatic diseases as well as medication for any of these. All participants scored normally on the German version of the CES-D Depression Scale (Radloff, 1977; Hautzinger and Bailer, 1993) and reported more positive than negative affect (positive affect: *M* = 18.8; SD = 6.2; negative affect: *M* = 3.6; SD = 3.3) on the PANAS (Watson et al., 1988) positive and negative affect scales.

Participants received course credit or were financially reimbursed for participation. The experimental procedure was conducted in accordance with the Declaration of Helsinki. Participants gave written informed consent prior to participation.

### Stimulus material

Seventy nouns were selected from a German word database (semantischer Atlas^1^) that provides for each word, ratings of valence, arousal, imageability, and concreteness. All selected nouns were of negative valence (*M* = 2.7, SD = 0.3), of moderate emotional arousal (*M* = 4.5, SD = 0.6), imageability (*M* = 5.0, SD = 0.96) and concreteness (*M* = 4.6, SD = 1.1), and stimuli were comparable in word length (*M* = 7.8 letters, SD = 2.2) and word frequency (*M* = 12.4, SD = 2.05). The stimulus material was grouped into 35 cue and 35 target words. Cue and target words were moderately semantically related (“Leipziger Korpus”^2^).

### Experimental procedure

Upon arrival, participants were informed about the experimental procedure in general terms. They gave written informed consent and filled in self-report questionnaires on mood and current affect.

### Learning phase

The Hint Training procedure (Anderson and Green, 2001) was used as the learning phase of the experiment. Prior to experimental testing, participants received all 35 cue-target pairs and were instructed to learn them in such a way that they would be able to remember the target word when the cue word was presented. Participants were free to use their best individual learning strategies (e.g., covering and un-covering the second word, learning the pairs in blocks, etc.). The time limit for the learning phase was 15 min and could be extended, if necessary, to ensure that each participant would reach a rate of more than 50% of correctly remembered target words prior to experimental intervention (Anderson and Green, 2001). Participants were informed that some of the cue words would either require a response or not and were asked to remember which of the cues would later require a response. Participants were given practice blocks to learn the cue-target associations. Rate of correctly learned word pairs was tested immediately after the learning phase. Cue words were presented on a computer screen for 4 s, participants were instructed to read the cue words and remember the related target word by speaking the target out aloud. The experimenter noted how many and which of the word pairs were correctly remembered to establish the baseline of individual memory performance prior to the intervention.

### Experimental intervention

The experimental intervention consisted of a modified version of the TNT paradigm. Participants were presented with the cue words from the learning phase (excluding the baseline words). Cue words were presented in two blocks. Each block consisted of the same 27 cue words: 18 cues were go/think and 9 cues were no-go/no-think items. Eight cue-target pairs were selected as baseline trials and not presented during the task. In each block, trials were presented in randomized order. Participants were instructed to press a response button with their right index finger as soon as a word that was previously learned as a go item appeared on the computer screen. They were also instructed to simultaneously remember the appropriate target word by speaking it out aloud. Thus, go trials demanded an overt verbal and behavioral response in addition to target word remembering. For no-go cues, participants were instructed to inhibit any overt response including remembering of the target word. Go and no-go items were presented for 1500 ms and followed by an inter-stimulus interval of 1000 ms. Participants had about 2.5 s to respond or inhibit their responses. Cue words were presented in black letters (font: Times 40) in the middle of the computer screen. The experiment was controlled by E-Prime 2.0 software (Psychology Software Tools, Inc., PA, USA).

### Recall phase

**Figure 1:**
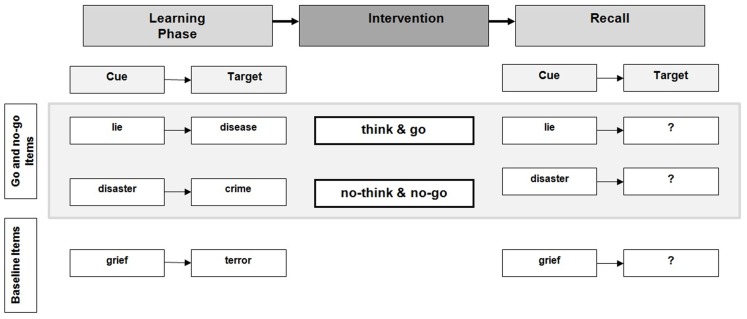
**Experimental design**.

Immediately after the intervention, participants performed a cued recall task. Cue words including baseline stimuli which were not shown in the TNT were presented on a computer screen in randomized order for 4 s each; participants were asked to recall as many of the target words as possible by speaking them out aloud. Correct and incorrect answers were recorded by the experimenter. A cued recall design instead of a cue-independent design (i.e., using new cues during the recall phase) was used to make the design compatible with previous TNT studies using emotional items (e.g., Anderson and Green, 2001; Depue et al., 2006, 2007) and to control for suppression related arousal effects. A detailed overview of the experiment is provided in Figure 1.

### Data reduction and statistical analysis

Percentage (%) of correctly processed trials (i.e., correctly processed go trials and successfully inhibited no-go trials) and mean reaction times of go trials were calculated. Memory performance (% of correctly remembered targets at pre- and post-intervention) was statistically evaluated by a repeated measures analysis of variance (ANOVA). The ANOVA included “target type” (go, no-go, baseline) and “time” (pre vs. post) as within subject factors. Significant main effects as well as interaction effects were decomposed by *post hoc* tests (Fisher’s LSD). Pre-post difference values (%) for each of the three target types (go, no-go, and baseline) were also analyzed in a separate one-way repeated measures ANOVA design containing the factor “target type” as the within subject factor. Significant main effects were again decomposed by *post hoc* tests (Fisher’s LSD).

## Results

### Reaction times and task performance

Participants responded correctly in 89.9% of the go trials. In these trials they responded to the cues by button press and speaking the target word aloud. Participants’ attempt to perform the dual-task correctly was also reflected in the reaction time data. The average response time for go trials was 1488 ms (SD = 445.13). For no-go trials, participants were able to inhibit any response in 89.1% of trials, which again indicates that participants performed the dual-task with high accuracy.

### Memory performance and accuracy (cued recall)

**Figure 2:**
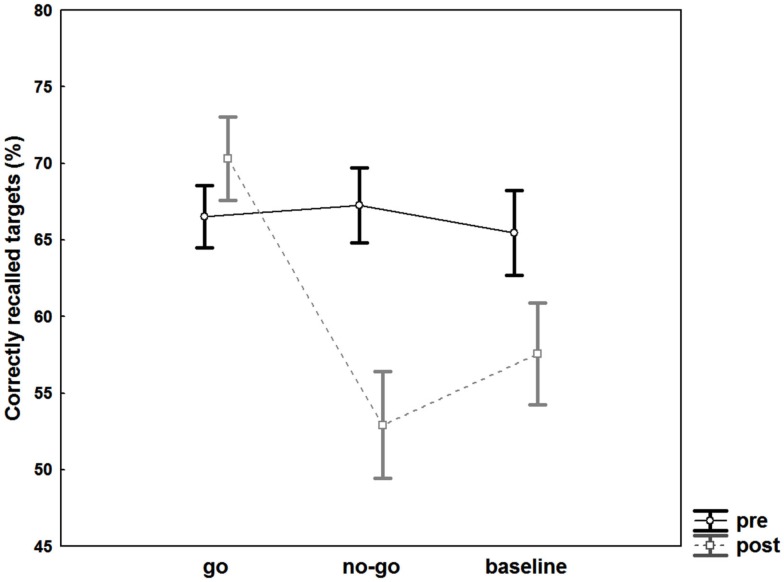
**Memory effects**. Recall performance of targets associated with go, no-go, and baseline cue words prior to (dark lines) and after intervention (gray lines). Error bars represent standard errors.

The ANOVA revealed significant main effects of the factors “target type” [*F*(2, 74) = 5.7, *p* = 0.005, η^2^ = 0.134] and “time” [*F*(1, 37) = 13.7, *p* < 0.001, η^2^ = 0.27] and a significant interaction effect of “target type × time” [*F*(2, 74) = 19.06, *p* < 0.001, η^2^ = 0.34]. *Post hoc* Fisher’s LSD tests revealed no difference in recall for go targets at post- compared to pre-testing [*t*(74) = 1.5, *p* = 0.07], but significant differences for no-go targets [*t*(74) = 5.2, *p* < 0.001] as well as baseline targets [*t*(74) = 3.5, *p* = 0.0003]. In addition, no-go targets were significantly less well remembered in comparison to go targets [*t*(74) = 5.2, *p* < 0.001] and baseline targets [*t*(74) = 1.9, *p* = 0.03] at post-testing. The ANOVA comparing pre-post difference values of go, no-go, and baseline targets revealed similar significant results for the main factor “target type” [*F*(2, 74) = 19.06, *p* < 0.001, η^2^ = 0.34] as the full factorial ANOVA design. Again, *post hoc* Fisher’s LSD tests showed significantly larger differences in memory performance for no-go targets compared to baseline targets [*t*(74) = 1.9, *p* = 0.03]. This suggests that memory suppression for no-go targets did exceed effects associated with forgetting over time (memory data for baseline words pre-post). ANOVA results are shown in Figure 2. Mean values for recalled targets (%) are presented in Table 1.

**Table 1:** **Mean values (%) of memory performance in all conditions**.

Trials	Correctly recalled targets (%)		
	Pre-test	Post-test	Difference
Go^a^	66.5 (2.04)	70.3 (2.73)	+3.8 (2.04)
No-go^a^	67.2 (2.44)	52.9 (3.49)	−14.3 (2.75)
Baseline^a^	65.5 (2.77)	57.6 (3.32)	−7.9 (2.33)

*^a^Standard errors are presented in parentheses*.

## Discussion

By combining the classical TNT and go/no-go paradigms this study investigated how doing the no-think and no-go tasks concurrently influences memory of unpleasant items during later recall. Instructions not to think and not to respond to the cues significantly reduced memory retrieval of targets that were previously learned to be associated with no-go/no-think cues. Memory impairment for no-go/no-think targets during later recall was found both in comparison to targets that participants were allowed to think of during go/think trials as well as in comparison to baseline targets that were not subject of any intervention at all.

The results contradict earlier studies predicting paradoxical effects of memory suppression. Regarding paradoxical effects, it has been argued that interventions that prompt individuals not to think and/or not to show any overt behavioral response maintain or even increase the accessibility of suppressed targets into conscious awareness, leading to ironic rebound effects at later retrieval (e.g., Wegner et al., 1987, 1990).

Likewise, it has been speculated that instructing subjects not to respond during no-think trials could act as a distracting task that consumes processing resources, which are then not available for memory inhibition (Román et al., 2009).

In contrast to this speculation, individuals of our study were able to accurately perform the two tasks. There were very few no-go/no-think and go/think trials with intrusions suggesting no competition of processing resources when TNT and motor response inhibition tasks are combined. Nevertheless, this does not mean that processes involved in thought suppression and response inhibition are unrelated. Neurocognitive studies demonstrated that on a neural level, processes involved in motor inhibition and memory suppression are closely related (Mecklinger et al., 2009; Depue et al., 2010), that memory suppression in the TNT activates prefrontal brain regions involved in motor inhibition (Anderson et al., 2004; Depue et al., 2007) and that activation of these prefrontal control regions is associated with decreased activity in memory structures such as the hippocampus (Anderson et al., 2004; Depue et al., 2007; Hanslmayr et al., 2009). Viewed from a neurophysiological perspective, it is likely that thought and response inhibition activate a common neuronal control system, which in turn exerts an inhibitory influence on subcortical memory structures such as the hippocampus leading to memory impairments for no-go/no-think targets during later recall.

Still, it could be argued that memory suppression effects during later recall are not the result of inhibitory processes. Tomlinson et al. (2009), for instance, suggested that effects in the TNT in general can result from memory interference during both stages of recall. The explanation by Tomlinson and colleagues offers a non-inhibitory explanation to results found in the TNT suggesting interference at the second (recovery) stage rather than just at the first sampling stage. However, this model has been debated (see Bäuml and Hanslmayr, 2010) and thus, as annotated by Huber et al. (2010), future studies are needed to scrutinize its assumptions.

It has been shown that memory suppression increases in strength with the number of times a word is inhibited during the TNT (e.g., Anderson and Green, 2001). Relatedly however, learning of new memory associations might increase with repeated suppression trials. In our study, only few no-go/no-think repetitions were used – perhaps not enough to establish strong new memory associations. This suggests that in the context of two inhibitory tasks including motor and thought suppression, memory suppression can be obtained even after a few intervention blocks.

Nevertheless, the results of the present study are challenged by some methodological limitations that could be improved in future studies. Our results demonstrate reduced recall of unwanted memories when suppression of unpleasant memory content on a cognitive level (i.e., attempts not to think) and inhibition of prepotent responses to these contents on a behavioral level (motor response inhibition) are combined. Both inhibitory tasks were performed equally well, suggesting complementary effects of motor response inhibition and memory inhibition, and no competition of cognitive processing resources. However, given that in the present study instructions not to think and not to respond were perfectly correlated, and no further control conditions were included in which participants performed one of the two tasks alone (no-think vs. not to respond), the present design is unable to determine the relative impact of response inhibition on memory suppression. Indeed, a fully balanced design including TNT conditions with and without response inhibition could extend the present findings and offer additional insight into how motor inhibition influences intentional thought suppression processes and how inhibitory effects spillover from one instruction to the other. Furthermore, memory tests using new cues during recall could be used to better understand the nature of inhibitory effects, i.e., if effects of response inhibition in the TNT are independent from associative priming effects or response biases related to the original cues. With this regard, however, care will have to be taken that cues used during the learning phase and the retrieval phase are matched for emotional arousal, because there is evidence that differences in stimulus arousal can influence suppression effects in the TNT (e.g., Depue et al., 2007; Marx et al., 2008).

In sum, the present study investigated inhibitory effects across modalities (thought and response inhibition) and studied their interaction (interference vs. complementary effects) on later memory. The results link directly to an increasingly relevant stream of research interested in the relationship between inhibitory processes involved in different tasks and processes. The findings strengthen previous speculations of cross-modality effects, supporting suggestions of a common inhibitory control system underlying thought and response control (e.g., Berkman et al., 2009; for review Aron, 2007).

## Conflict of Interest Statement

The authors declare that the research was conducted in the absence of any commercial or financial relationships that could be construed as a potential conflict of interest.

## Footnotes

^1^http://dpz.eu/web/docs/semat/semat.html

^2^www.wortschatz.uni-leipzig.de
